# Modular Hemipelvic Prosthesis Preserves Normal Biomechanics and Showed Good Compatibility: A Finite Element Analysis

**DOI:** 10.3390/jfb15090276

**Published:** 2024-09-21

**Authors:** Yuanrui Luo, Hongtao Sheng, Yong Zhou, Li Min, Chongqi Tu, Yi Luo

**Affiliations:** 1 Department of Orthopedics, Orthopedic Research Institute, West China Hospital, Sichuan University, Chengdu 610041, China; luoyuanrui2021@163.com (Y.L.); shenghongtaogd@163.com (H.S.); zhouyonggk@163.com (Y.Z.); jacky-min@163.com (L.M.); tucq@scu.edu.cn (C.T.); 2 Model Worker and Craftsman Talent Innovation Workshop of Sichuan Province, No. 37 Guoxue Road, Chengdu 610041, China

**Keywords:** hemipelvic prosthesis, biomechanics, stress distribution, three-dimensional finite element, center of rotation

## Abstract

This study aimed to evaluate the biomechanical compatibility of a modular hemipelvic prosthesis by comparing stress distributions between an implanted pelvis and a healthy pelvis. Finite element analysis was used to simulate bilateral standing loads on both models, analyzing critical regions such as the sacroiliac joints, iliac crest, acetabulum, and prosthesis connection points. Six models with varied displacements of the hip joint rotational center were also introduced to assess the impact of deviations on stress distribution. The implanted pelvis had a stress distribution closely matching that of the intact pelvis, indicating that the prosthesis design maintained the biomechanical integrity of the pelvis. Stress patterns in displacement models with deviations of less than 10 mm were similar to the standard model, with only minor changes in stress magnitude. However, backward, upward, and inward deviations resulted in stress concentrations, particularly in the prosthesis connection points, increasing the likelihood of mechanical failure. The modular hemipelvic prosthesis demonstrated good biomechanical compatibility with minimal impact on pelvic stress distribution, even with moderate deviations in the hip joint’s rotational center; outward, forward, and downward displacements are preferable to minimize stress concentration and prevent implant failure in cases where minor deviations in the rotational center are unavoidable during surgery.

## 1. Introduction

With advancements in computer technology, finite element analysis (FEA) has become a crucial method in biomechanics research and is now widely applied in the medical field [1,2,3]. Its applications are primarily concentrated in two areas: (1) Design and optimization of medical devices [4,5,6]: the mechanical properties of medical devices often determine their clinical effectiveness. Using FEA to simulate mechanical experiments on devices offers advantages such as shorter research times, lower costs, comprehensive mechanical performance testing, and strong repeatability. This method aids in both the design and improvement of medical devices. (2) Biomechanical simulation experiments [7,8,9]: by establishing three-dimensional finite element models of the human body, including bones, blood vessels, muscles, and other vital organs, and assigning biomechanical properties to these models, researchers can conduct mechanical experiments such as tension, bending, torsion, and fatigue resistance. These experiments analyze deformation, stress distribution, strain distribution, internal energy changes, and ultimate failure under different experimental conditions. FEA has already been extensively applied to pelvic biomechanics research [10,11,12,13,14,15].

The pelvis is one of the common sites for primary and metastatic tumors, with from approximately 10% to 15% of primary malignant bone tumors occurring in the pelvis [16]. Pelvic tumor resection and reconstruction, especially around the acetabulum, are challenging procedures with high technical demands and many postoperative complications. Currently, due to advancements in adjuvant chemotherapy, imaging, and surgical techniques, limb-salvage surgery has become the primary treatment method for malignant pelvic tumors [17]. Surgical reconstruction options after tumor resection include arthrodesis, hip transposition, allograft/autograft reconstruction, and endoprosthetic reconstruction [18,19,20,21,22,23]. Among these, endoprosthetic reconstruction is favored for its stability, aesthetic benefits, early mobility, and the absence of complications related to bone grafting. In clinical settings, various types of hemipelvic endoprostheses are employed, such as ice-cream cone prostheses, saddle prostheses, modular prostheses, and 3D-printed hemipelvic prostheses [24,25,26,27,28,29,30]. Ice-cream cone and saddle prostheses, however, require substantial retention of the ilium for fixation, which restricts their use in many cases [31]. Moreover, 3D-printed hemipelvic prostheses offer several advantages, including a highly customized fit that improves anatomical matching and allows for better reconstruction of the pelvis. They facilitate personalized surgical approaches, which can enhance postoperative function and patient outcomes such as reducing the risk of infection, the chance of dislocation, or failure of the implant. Additionally, 3D printing enables complex designs that are difficult to achieve with traditional manufacturing methods. However, these prostheses also come with notable limitations, such as long production times, high costs, and limited long-term clinical data. Challenges with osteointegration and variability in mechanical properties further impact their durability and overall performance, making them less suitable for urgent or cost-sensitive cases [29,32,33]. Modular hemipelvic prostheses offer the flexibility to be assembled intraoperatively, even when significant iliac resection is necessary [16,34]. Their relatively smaller size allows for better soft tissue coverage during surgery, reducing dead space and enabling stronger muscle reconstruction, which enhances hip joint function rehabilitation and theoretically lowers the risk of deep postoperative infections. Early studies on modular hemipelvic prosthesis replacement for pelvic ring defects have shown that stress is primarily distributed in the sacroiliac joint, arcuate line, acetabulum, and femoral neck, aligning with the path of pelvic force conduction [35,36,37]. Postoperative patients can engage in necessary physical activities early on, meeting partial weight-bearing needs.

However, due to varying degrees of pelvic ring defects following pelvic tumor resection, it is difficult to accurately position the acetabulum during prosthesis placement, leading to potential positional deviations and displacements in the hip joint rotation center. The impact of these deviations on pelvic stress distribution after modular hemipelvic prosthesis replacement has not yet been reported. This study uses thin-slice CT scan data to establish finite element models of different hip joint rotation centers following modular hemipelvic prosthesis replacement. By simulating stress loading in a standing position, we analyze various models’ stress magnitude and distribution to provide biomechanical guidance for clinical practice.

## 2. Materials and Methods

### 2.1. Experimental Materials and Equipment

#### 2.1.1. Prosthetic Materials

**Figure 1:**
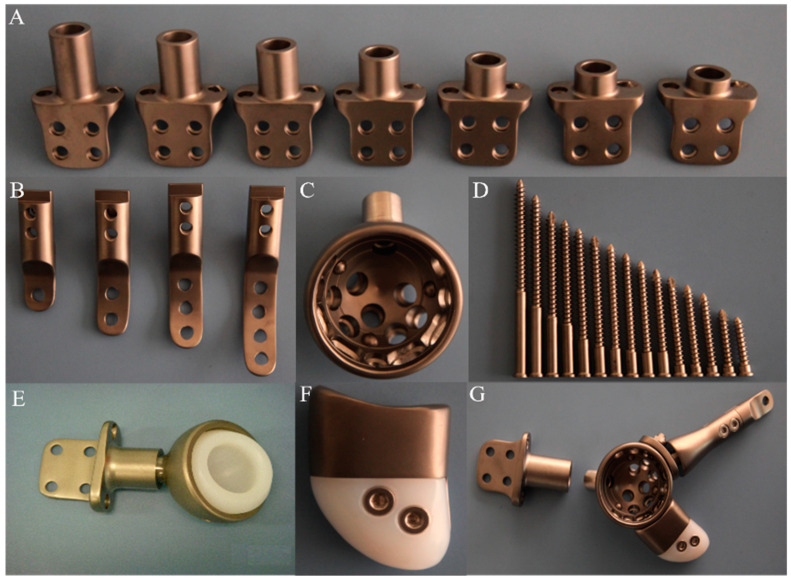
Composition of the modular semi-pelvic prosthesis: (**A**) CS spinal internal fixation device; (**B**) pubic branch; (**C**) acetabular cup; (**D**) fixation screws; (**E**) spinal internal fixation device, acetabular cup, and inner cup; (**F**) ischial branch; (**G**) assembly diagram of the hemipelvic prosthesis.

The modular hemipelvic prosthesis was provided by Beijing Chunli Zhengda Company (Figure 1). The prosthesis was made of titanium alloy with a smooth surface. The acetabular cup had an outer diameter of 54 mm and an inner diameter of 50 mm. The CS (CHUNLI SYSTEM) spinal internal fixation device had a consistent base size, with connection rods of varying lengths to adjust the distance from the base to the acetabular cup. The pubic branch also came in various lengths and models for selection.

#### 2.1.2. Hardware Equipment

A spiral CT scanner (Philips Brilliance 64, Philips Company, Amsterdam, The Netherlands), provided by the Radiology Center of West China Hospital, Sichuan University, was used for three-dimensional CT scans of normal pelvis. A computer (Windows 7; CPU Core II E6300 2.4 GHz; 4 GB DDR RAM; 512 MB VRAM; 360 GB HDD), provided by the Biomechanics Center of Sichuan University, was used for modeling, mechanical loading, and data computation.

#### 2.1.3. Analysis Software

Modeling software included Mimics 19.0 (Materialise Company, Leuven, Belgium) for creating three-dimensional solid models. SolidWorks 2021 was used to generate solid models of the modular hemipelvic prosthesis components and construct standard and different hip joint rotation center models after tumor resection and prosthesis replacement. The models were converted via Proe Wildfire 5.0 and imported into ANSYS 19.0 for three-dimensional finite element analysis.

### 2.2. Establishing the Three-Dimensional Finite Element Model

#### 2.2.1. Data Collection

**Figure 2:**
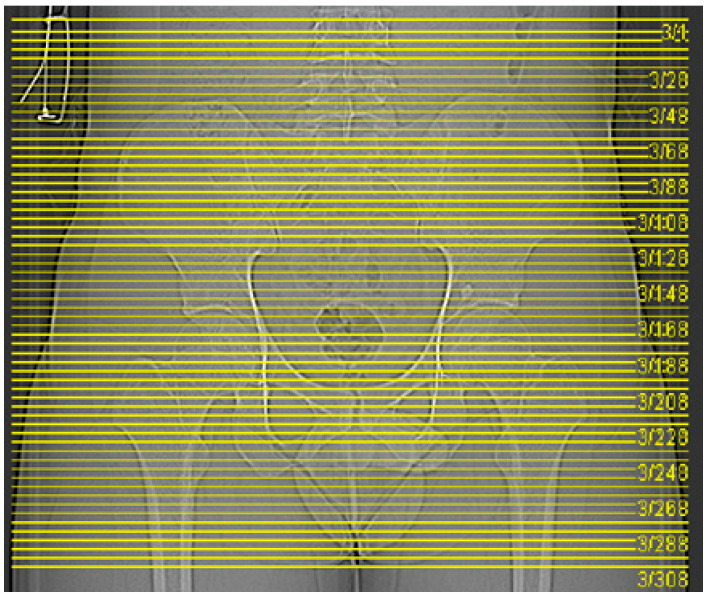
A pelvic CT scan of a healthy adult.

Data were collected from a healthy 26-year-old male volunteer, 174 cm tall and weighing 70 kg. The pelvis and lower limbs were positioned in a neutral rotational position for spiral three-dimensional CT scanning along the body’s long axis, from the L4 level to the mid-femur level (Figure 2). Scanning parameters were set using the spine scanning sequence: 120 KV, Total mAs of 1328 mA, Total DLP of 227 mGycm, scanning 894 slices with a layer thickness of 0.6 mm, covering a total of 536.4 mm, and obtaining DICOM data.

#### 2.2.2. Three-Dimensional Reconstruction of Pelvic

**Figure 3:**
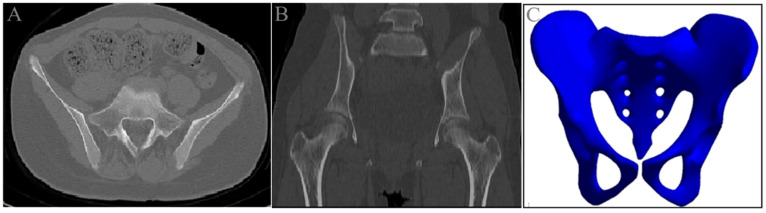
Three-dimensional reconstruction of the pelvis. (**A**) Pelvic cross-sectional CT scan; (**B**) pelvic coronal CT scan; (**C**) 3D reconstruction model of the normal pelvis.

The CT data were exported as DICOM images and imported into Mimics 19.0 software. Based on the differences in CT image grayscale values, the images were defined as cancellous bone, cortical bone, marrow cavity tissue, and muscle soft tissue. The skeletal data were extracted from the DICOM files, selecting a region of interest of 416.0 mm (with the upper boundary at the S1 vertebral body and the lower boundary at the ischial tuberosity, excluding the femoral head, with 693 slices scanned). This process was used for the three-dimensional reconstruction of the normal pelvis (Figure 3).

#### 2.2.3. Normal Pelvis and Solid Model

**Figure 4:**
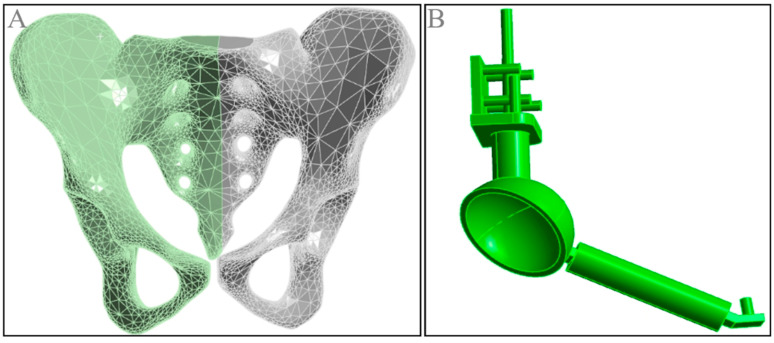
Solid model of pelvis and prosthesis. (**A**) Solid model of the normal pelvis; (**B**) solid model of the modular hemipelvic prosthesis.

The three-dimensional reconstruction data from Mimics were imported into SolidWorks 2021 software to construct the solid model of the normal pelvis. Prosthetic data, provided by Beijing Chunli Zhengda Company (Beijing, China), included a complete set of prosthetic components. After measuring the relevant data, the solid model of the prosthesis was established in SolidWorks 2021 (Figure 4).

#### 2.2.4. Pelvic Defect Solid Model

**Figure 5:**
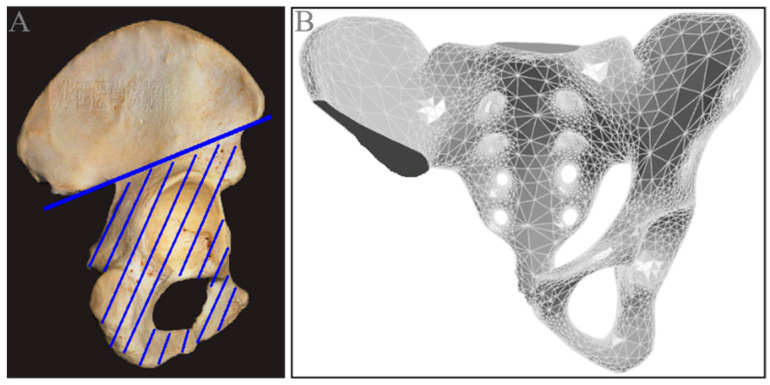
Establishment of the solid model of the pelvic defect. (**A**) Schematic diagram of the extent of the pelvic resection (the blue line); (**B**) solid model of the pelvic defect.

Based on the solid model of the normal pelvis, a pelvic defect solid model was constructed in SolidWorks 2021 by simulating tumor resection around the acetabulum (Figure 5). Drawing on previous research experience [36], the scope of resection was from the lower margin of the anterior superior spine of the iliac to the greater ischiatic notch, and the pubic bone and ischiatic bone on the affected side were removed as a whole from the symphysis pubis.

#### 2.2.5. Standard Model and Rotation Center Displacement Model

**Figure 6:**
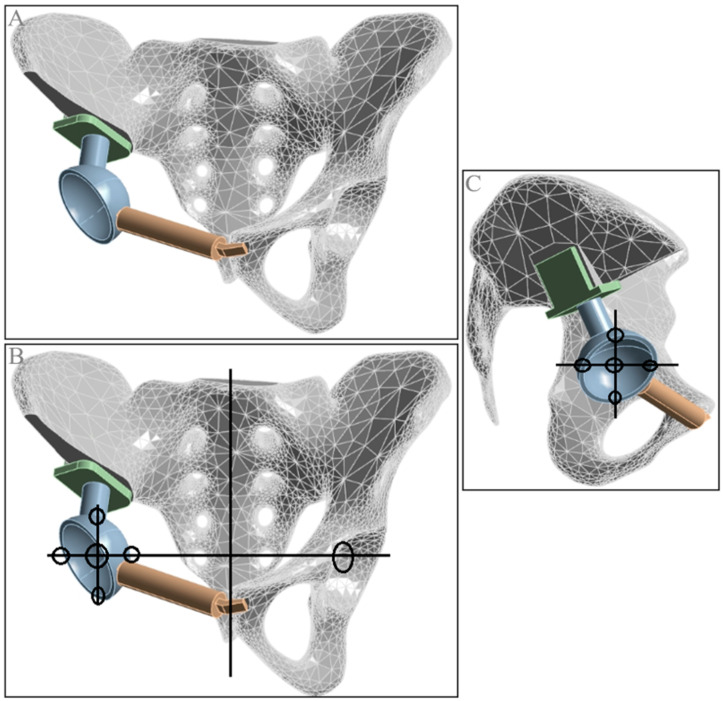
Hemipelvic prosthesis standard position solid model and rotation center displacement diagram. (**A**) Standard model; (**B**) lateral and vertical displacement diagram; (**C**) vertical and anterior–posterior displacement diagram.

Using SolidWorks 2021 software, a three-dimensional pelvic defect model was imported to simulate hemipelvectomy and modular prosthesis assembly, creating the solid prosthesis assembly model. To establish the standard (position) solid model, the normal side of the pelvis was used as a reference, aligning the top of the prosthetic acetabulum symmetrically with the normal acetabulum across the mid-sagittal plane. The acetabular abduction and anteversion angles were matched to those of the normal side. An appropriate spinal internal fixation device was selected to fit the residual iliac bone cross-section, secured laterally to the iliac bone with four screws and centrally with a longer screw through the top of the prosthetic acetabulum and the spinal fixation device. A suitably long pubic branch was chosen to connect the prosthetic acetabulum to the contralateral superior pubic ramus, fixed with two screws (Figure 6).

**Figure 7:**
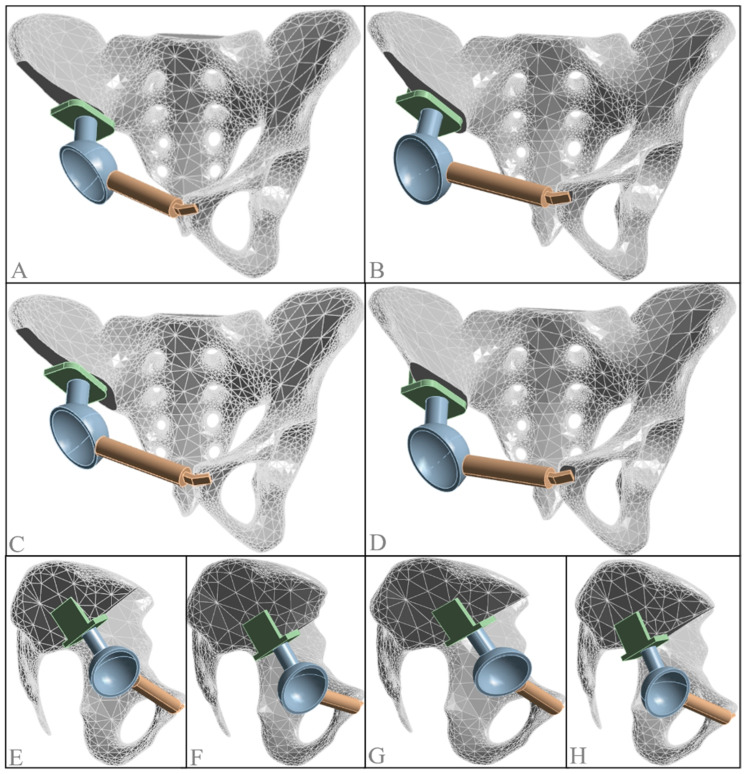
Models of different hip joint rotation centers after prosthetic replacement surgery. (**A**) Inward displacement model; (**B**) outward displacement model; (**C**) upward displacement model ((**E**) lateral view); (**D**) downward displacement model ((**F**) lateral view); (**G**) forward displacement model; (**H**) backward displacement model.

When establishing solid models with different hip joint rotation centers, the standard solid model was used as the baseline, with all models maintaining the same acetabular abduction and anteversion angles as the contralateral side. Displacement occurred along a single axis, with a displacement distance of 10 mm. For instance, in the outward displacement (10 mm) model, the acetabular abduction and anteversion angles were consistent with the normal side. The acetabular center was aligned in the anteroposterior and vertical directions but displaced outward by 10 mm from the standard model. Similarly, models with 10 mm inward, downward, upward, forward, and backward displacements were constructed (Figure 7).

#### 2.2.6. Material Parameters and 3D Finite Element Mesh Generation

Based on the various solid models constructed using SolidWorks 2021, seamless integration was achieved through Proe Wildfire 5.0 and ANSYS 19.0 software. In calculating compressive stress distribution, Poisson’s ratio and elastic modulus are the primary influencing factors [38]. Poisson’s ratio, also known as the transverse deformation coefficient, reflects the lateral deformation properties of a material. It is defined as the absolute value of the ratio of transverse strain (ε1) to longitudinal strain (ε2) under uniform longitudinal stress. The elastic modulus, or Young’s modulus, represents the proportional relationship between stress and strain during material deformation. The material parameters used in this study were provided by Beijing Chunlizhengda Medical Instruments Co., Ltd. (Beijing, China) and included titanium plates (pubic plates) and titanium alloy rods (CS spinal internal fixators, acetabular cup connectors).

Due to the complexity of the model system, relevant material parameters and boundary conditions were set for finite element calculations to facilitate model establishment [11,39,40,41,42]. The following settings were applied: (1) both the prosthesis and bone were modeled as isotropic continuous elastic materials, and (2) the interfaces between them were fully bonded.

The finite element model in this study was configured as follows: each component of the model was treated as a continuous, homogeneous, isotropic linear elastic material. The material properties of the prosthesis were assigned based on previous research (Table 1).

**Figure 8:**
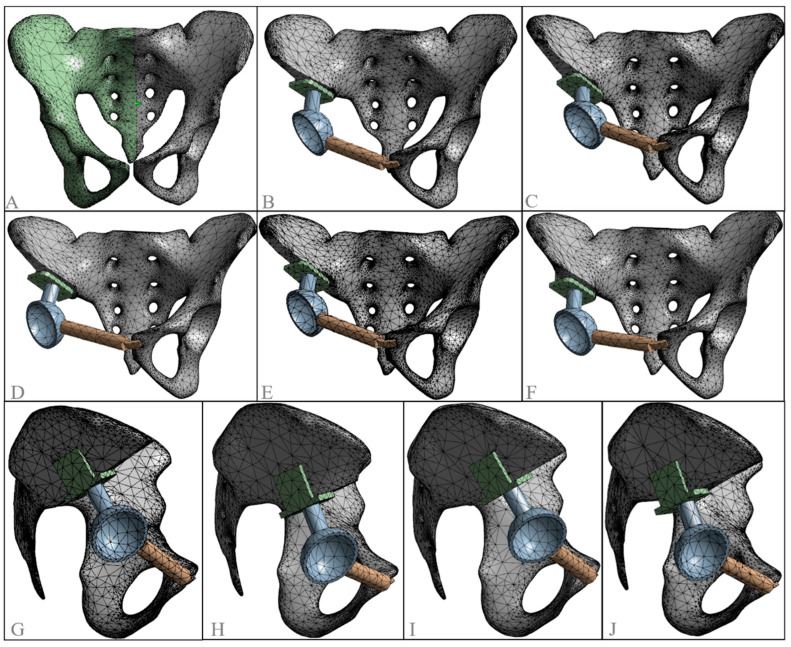
Three-dimensional finite element meshed models. (**A**) normal model; (**B**) standard model; (**C**) inward displacement model; (**D**) outward displacement model; (**E**) upward displacement model; (**F**) downward displacement model; (**G**) lateral view of the outward displacement model; (**H**) lateral view of the upward displacement model; (**I**) lateral view of the forward displacement model; (**J**) lateral view of the backward displacement model.

In ANSYS 19.0 software, the first-order tetrahedral element [43] was selected for meshing the model. This process was applied to both the normal pelvis and the reconstructed hemipelvis with the prosthesis, creating different 3D finite element models with varying rotation centers (Figure 8). The model was divided into several element units, as outlined in Table 2.

### 2.3. Mechanical Loading and Computation

#### 2.3.1. Mechanical Loading

Previous researchers have incorporated the attachment of surrounding muscles into three-dimensional finite element modeling to analyze stress distribution in the pelvis comprehensively. This approach involves connecting the muscles’ physiological attachment points at their proximal and distal ends to determine the direction of muscle contraction forces [41,44,45,46]. However, this study primarily focused on analyzing stress distribution in the pelvic ring after various types of modular hemipelvic prosthesis replacement without fully considering the acetabulum and its lower structures or the impact of peri-acetabular muscles on pelvic biomechanics.

Using ANSYS 19.0 for three-dimensional finite element analysis, we simulated the standard standing position of the patient’s lower limbs. The degrees of freedom at the nodes on the lower ends of the femurs were constrained in all directions using the ‘Fix to current’ method. Mechanical loading was then applied to both the normal pelvic model and the models with different hip joint rotation centers. Given that the upper body accounts for approximately two-thirds of a person’s total weight, the hip joints bear an equivalent load during bipedal standing. In a single-leg stance, the hip joint on the standing side supports about 81% of the body weight. During walking, pelvic stress can reach 4–7 times the body weight, depending on the gait phase (with the heel-strike side bearing around 4 times and the toe-off side up to 7 times the body weight). These forces increase with walking speed, peaking during activities like running and jumping, where stress may reach up to 10 times the body weight.

**Figure 9:**
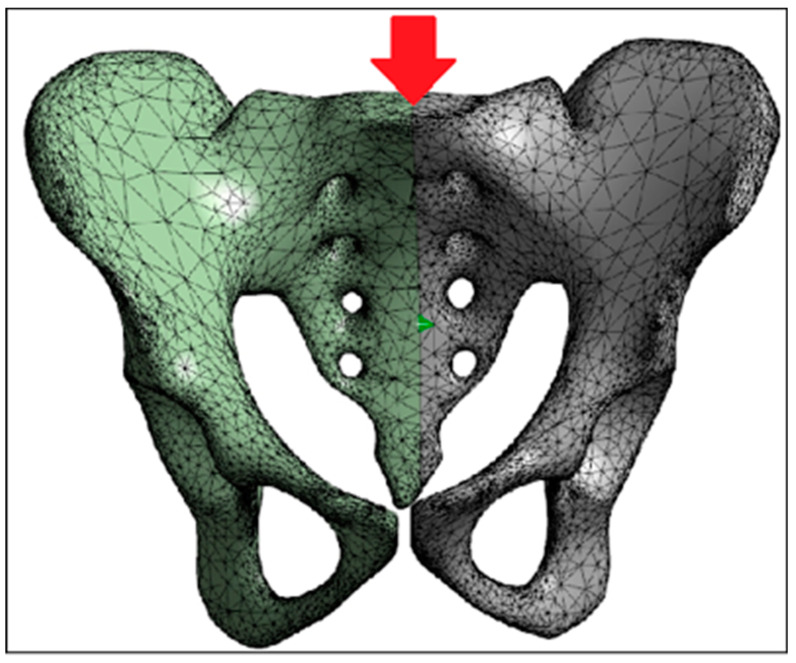
Schematic diagram of mechanical loading direction.

In this study, the volunteer weighed 70 kg, and we focused on pelvic biomechanics during bipedal standing. Based on previous research [36,37], a loading force equivalent to the volunteer’s body weight (700 N) was applied. A vertical downward force was directed along the center of the S1 vertebra, with constraints applied to the tops of both acetabula (Figure 9).

#### 2.3.2. Stress Analysis

**Figure 10:**
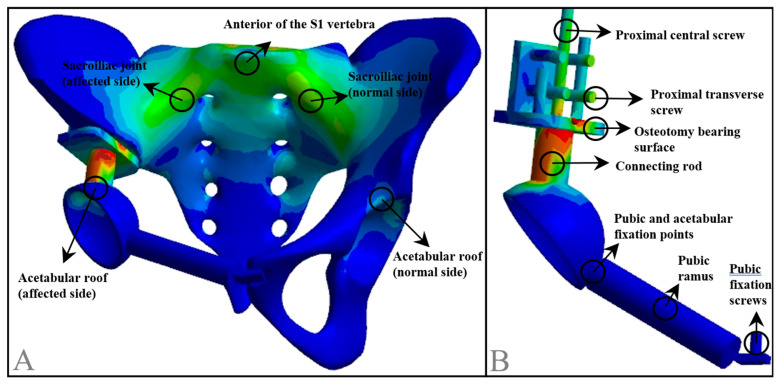
Schematic diagram of stress measurement locations in the pelvis and prosthesis model. (**A**) Stress measurement points in the pelvic model; (**B**) stress measurement points in the prosthesis model.

The stress distribution and magnitude in various finite element models were analyzed, focusing on the anterior portion of the S1 vertebra, bilateral sacroiliac joints, and the acetabular roof. For the prosthesis, the analysis of stress distribution and magnitude primarily included the proximal central screw, proximal transverse screw, osteotomy bearing surface, connecting rod, pubic and acetabular fixation points, pubic ramus, and pubic fixation screws (Figure 10).

## 3. Results

### 3.1. Stress Distribution in a Three-Dimensional Finite Element Model of a Normal Pelvis

**Figure 11:**
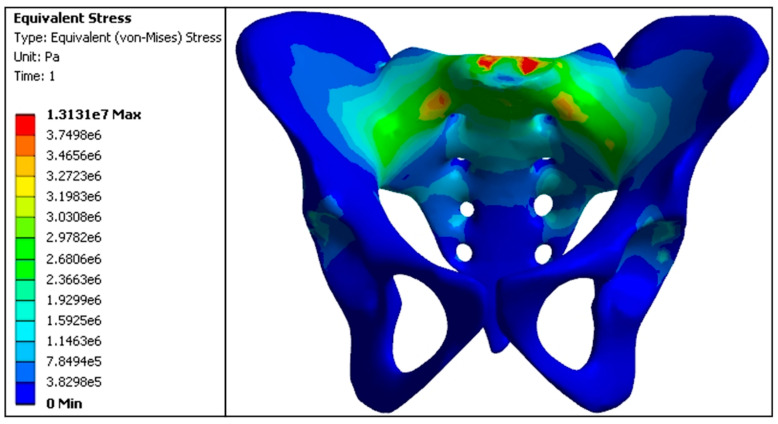
Stress distribution nephogram of the standing position of both feet in a normal pelvis model (e5 = × 10^5^, e6 = × 10^6^, e7 = × 10^7^).

In the normal pelvic model, stress was transmitted through the S1 vertebral body, bilateral sacroiliac joints, and pubic symphysis to the acetabular roof and both lower limbs. The maximum stress values were 3.24 MPa at the anterior part of the S1 vertebral body, 3.50 MPa at the sacroiliac joints, 1.71 MPa at the acetabular roof, and 0.0050 MPa at the pubic ramus, with a symmetric distribution on both sides of the pelvis (Figure 11 and Table 3).

### 3.2. Finite Element Stress Distribution in the Standard Model

**Figure 12:**
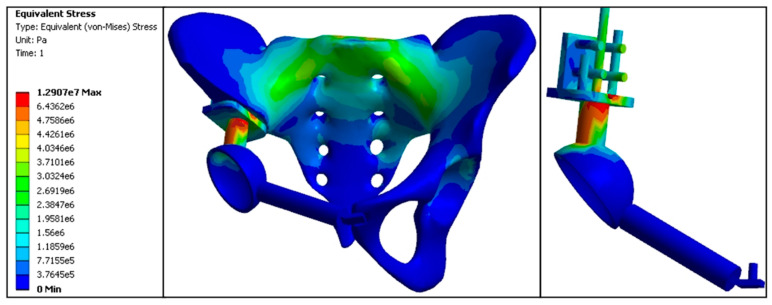
Standard model finite element stress distribution nephogram (e5 = × 10^5^, e6 = × 10^6^, e7 = × 10^7^).

In the standard model, pelvic stress was transmitted from the anterior S1 vertebra, bilateral sacroiliac joints, and arcuate line to the acetabular roof. The maximum stress values were as follows: 3.15 MPa at the anterior S1 vertebra, with the affected side showing higher stress at the sacroiliac joint (3.08 MPa) compared to the normal side (3.04 MPa), and at the acetabular roof (3.02 MPa) compared to the normal side (1.63 MPa). The proximal central screw experienced 3.11 MPa, the proximal transverse screw experienced 3.20 MPa, the cortical bone bearing surface experienced 6.48 MPa, and the connecting rod experienced 7.26 MPa, marking the highest stress areas in the model. Stress at the fixation points of the pubis and acetabulum was 0.0028 MPa; at the pubic ramus, 0.0019 MPa; and at the pubic fixation screw, 0.0035 MPa. Overall, the stress distribution in the standard model prosthesis was relatively uniform, similar to that of the normal pelvic model. The stress values at the pubic ramus, acetabulum fixation points, and pubic fixation screw were minimal compared to all replacement models (Figure 12, Table 4 and Table 5).

### 3.3. Finite Element Stress Distribution in Models with the Displacement Rotation Center

#### 3.3.1. Stress Distribution in the Inward Displacement Model

**Figure 13:**
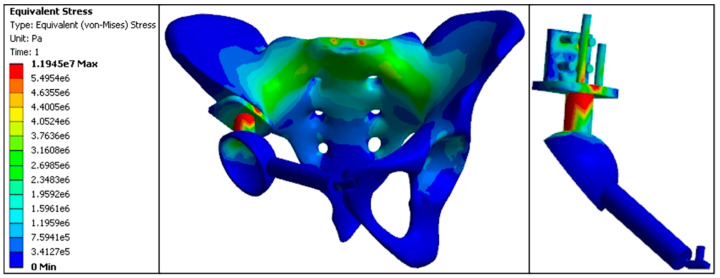
Finite element stress distribution nephogram of the inward displacement model (e5 = × 10^5^, e6 = × 10^6^, e7 = × 10^7^).

The maximum stress value at the anterior S1 vertebra was 3.56 MPa. Stress at the sacroiliac joint was lower on the affected side (3.31 MPa) compared to the normal side (3.47 MPa). In comparison, stress at the acetabular roof was higher on the affected side (3.20 MPa) compared to the normal side (1.43 MPa). The proximal central screw experienced a stress of 4.96 MPa, the proximal transverse screw experienced 2.39 MPa, the cortical bone bearing surface experienced 4.94 MPa, and the connecting rod experienced 8.61 MPa, with the connecting rod representing the highest stress area in this model. Stress values at the fixation points of the pubis and acetabulum were 0.0676 MPa; at the pubic ramus, 0.0484 MPa; and the pubic fixation screw, 0.0513 MPa. Overall, the stress distribution in the inward displacement model prosthesis was relatively uniform, with the highest stress values observed at the pubic ramus, acetabulum fixation points, and the pubic fixation screw. This indicates that the inward displacement model was prone to stress concentration at the pubic ramus, resulting in elevated stress values (Figure 13, Table 5 and Table 6)

#### 3.3.2. Stress Distribution in the Outward Displacement Model

**Figure 14:**
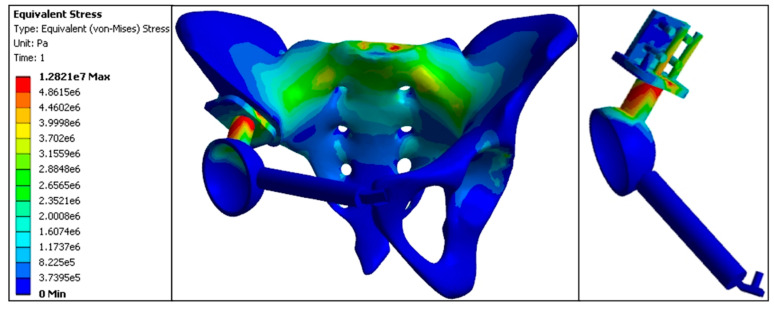
Finite element stress distribution nephogram of the outward displacement model (e5 = × 10^5^, e6 = × 10^6^, e7 = × 10^7^).

The maximum stress value at the anterior S1 vertebra was 3.19 MPa. Stress at the sacroiliac joint was lower on the affected side (3.27 MPa) compared to the normal side (3.51 MPa). In comparison, stress at the acetabular roof was higher on the affected side (2.69 MPa) compared to the normal side (1.73 MPa). The proximal central screw experienced a stress of 4.38 MPa, the proximal transverse screw experienced 4.44 MPa, the cortical bone bearing surface experienced 4.06 MPa, and the connecting rod experienced 5.36 MPa, with the connecting rod representing the highest stress area in this model. However, stress values at the cortical bone-bearing surface and the connecting rod were lower than those in the standard model. Stress values at the fixation points of the pubis and acetabulum were 0.0077 MPa; at the pubic ramus, 0.0060 MPa; and the pubic fixation screw, 0.0074 MPa. Overall, the stress distribution in the outward displacement model was more uniform compared to the inward displacement model. Except for the proximal transverse screw, which showed higher stress compared to the inward displacement model, other locations exhibited lower stress. This suggests that in the horizontal direction, the stress distribution and magnitude in the outward displacement model were closer to those of the standard model, with the pelvis and prosthesis showing a more uniform stress distribution and lower stress values compared to the inward displacement model (Figure 14, Table 5 and Table 7).

#### 3.3.3. Stress Distribution in the Backward Displacement Model

**Figure 15:**
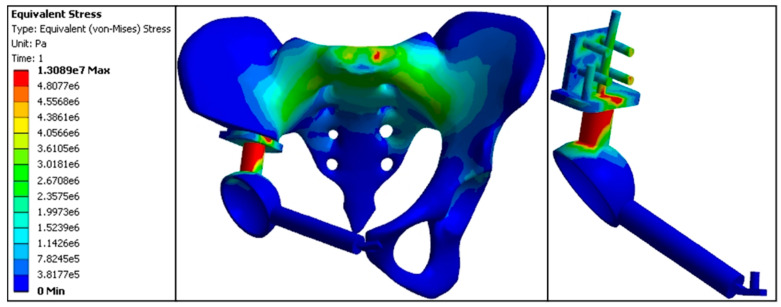
Finite element stress distribution nephogram of the backward model (e5 = × 10^5^, e6 = × 10^6^, e7 = × 10^7^).

The maximum stress value at the anterior S1 vertebra was 3.08 MPa. Stress at the sacroiliac joint was higher on the affected side (3.30 MPa) compared to the normal side (3.20 MPa). Stress at the acetabular roof was also higher on the affected side (3.31 MPa) compared to the normal side (2.10 MPa). The proximal central screw experienced a stress of 4.45 MPa, the proximal transverse screw experienced 4.06 MPa, the cortical bone bearing surface experienced 5.32 MPa, and the connecting rod experienced 9.31 MPa, with the connecting rod representing the highest stress area in this model. Stress values at the fixation points of the pubis and acetabulum were 0.0132 MPa; at the pubic ramus, 0.0091 MPa; and the pubic fixation screw, 0.0104 MPa. Notably, stress at the proximal and distal connection points of the prosthesis was significantly increased compared to the standard replacement model, particularly at the proximal transverse fixation screw and the connecting rod, which exhibited the highest stress values among all models. This indicates that the backward displacement model was prone to stress concentration at the affected side sacroiliac joint, connecting rod, and proximal transverse screw (Figure 15, Table 5 and Table 8).

#### 3.3.4. Stress Distribution in the Forward Displacement Model

**Figure 16:**
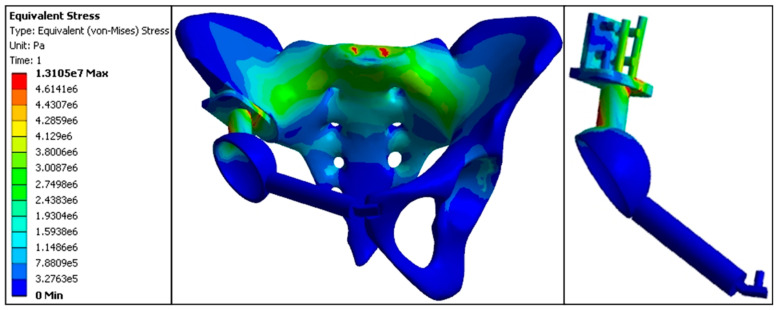
Finite element stress distribution nephogram of the forward model (e5 = × 10^5^, e6 = × 10^6^, e7 = × 10^7^).

The maximum stress value at the anterior S1 vertebra was 3.29 MPa. Stress at the sacroiliac joint was lower on the affected side (3.37 MPa) compared to the normal side (3.48 MPa). In comparison, stress at the acetabular roof was higher on the affected side (2.94 MPa) compared to the normal side (1.78 MPa). The proximal central screw experienced a stress of 4.05 MPa, the proximal transverse screw experienced 3.23 MPa, the cortical bone bearing surface experienced 5.13 MPa, and the connecting rod experienced 7.81 MPa, with the connecting rod representing the highest stress area in this model. Stress values at the fixation points of the pubis and acetabulum were 0.0089 MPa; at the pubic ramus, 0.0076 MPa; and the pubic fixation screw, 0.0103 MPa. The stress distribution in the forward displacement model was more uniform, with less noticeable stress concentration. Compared to the backward displacement model, the stress distribution and magnitudes in the forward displacement model were closer to those of the standard model, which helped to avoid stress concentration and resulted in overall lower stress values (Figure 16, Table 5 and Table 9).

#### 3.3.5. Stress Distribution in the upward Displacement Model

**Figure 17:**
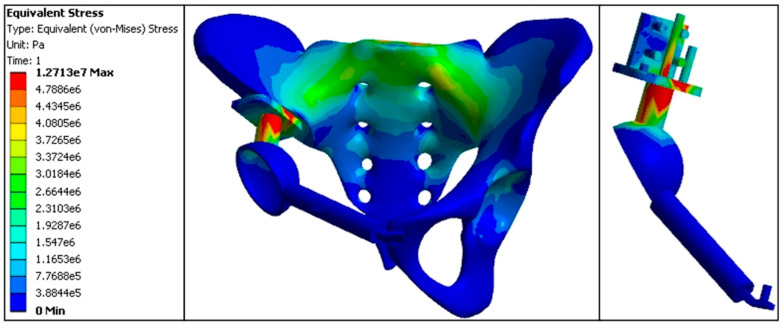
Finite element stress distribution nephogram of the upward model (e5 = × 10^5^, e6 = × 10^6^, e7 = × 10^7^).

The maximum stress value at the anterior S1 vertebra was 3.25 MPa. Stress at the sacroiliac joint was lower on the affected side (3.14 MPa) compared to the normal side (3.60 MPa). In comparison, stress at the acetabular roof was higher on the affected side (3.49 MPa) compared to the normal side (1.52 MPa). The proximal central screw experienced a stress of 5.31 MPa, the proximal transverse screw experienced 2.63 MPa, the cortical bone bearing surface experienced 7.14 MPa, and the connecting rod experienced 8.28 MPa, with the connecting rod representing the highest stress area in this model. Stress values at the fixation points of the pubis and acetabulum were 0.0305 MPa; at the pubic ramus, 0.0085 MPa; and the pubic fixation screw, 0.0191 MPa. Notably, the stress values at the proximal central screw and the cortical bone-bearing surface were the highest among all models, indicating that stress concentration was likely to occur at the bearing surface of the spinal internal fixation prosthesis in this model (Figure 17, Table 5 and Table 10).

#### 3.3.6. Stress Distribution in the Downward Displacement Model

**Figure 18:**
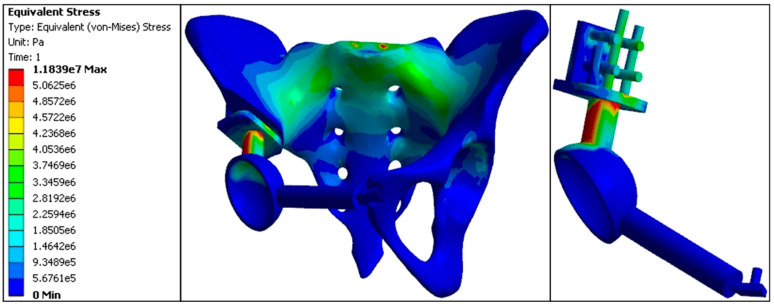
Finite element stress distribution nephogram of the downward model (e5 = × 10^5^, e6 = × 10^6^, e7 = × 10^7^).

The maximum stress value at the anterior S1 vertebra was 3.27 MPa. Stress at the sacroiliac joint was lower on the affected side (3.15 MPa) compared to the normal side (3.43 MPa), while stress at the acetabular roof was higher on the affected side (2.86 MPa) compared to the normal side (1.60 MPa). The proximal central screw experienced a stress of 3.65 MPa, the proximal transverse screw experienced 2.82 MPa, the cortical bone bearing surface experienced 3.98 MPa, and the connecting rod experienced 5.91 MPa, with the connecting rod representing the highest stress area in this model. Stress values at the fixation points of the pubis and acetabulum were 0.0075 MPa; at the pubic ramus, 0.0029 MPa; and the pubic fixation screw, 0.0101 MPa. Stress values for the prosthesis connecting rod and cortical bone-bearing surface were lower than those in the standard model, and the stress distribution in other areas was more uniform. Compared to the upward displacement model, the downward displacement model exhibited lower stress values. This indicates that, in the vertical direction, the downward displacement model had a more uniform stress distribution, which helped to avoid stress concentration and resulted in lower overall stress values (Figure 18, Table 5 and Table 11).

## 4. Discussion

Due to variations in pelvic shape and size among different genders, races, and regions, researchers have utilized diverse subjects for three-dimensional finite element modeling of the pelvis [40,47]. The complexity of the pelvic ring’s anatomical structure makes it challenging to establish an accurate and robust research model through anatomical measurement, sketching, and data imported into CAD (Computer-Aided Design) software [48]. Currently, CT and MRI cross-sectional imaging data are frequently used, with CT image stacking technology employed to reconstruct three-dimensional models [49,50,51,52]. The thickness, resolution, and reconstruction methods of cross-sectional images directly influence model accuracy. Higher precision leads to experimental results that more accurately reflect the human body’s actual situation. Micro-computed tomography (Micro-CT) offers detailed bone microstructure data for three-dimensional finite element modeling and allows for precise stress and strain analysis of cancellous bone, callus, and surrounding soft tissue. Consequently, some researchers have used micro-CT scanning data for modeling, significantly enhancing the accuracy of the models and experimental results [53,54]. In this study, pelvic data were obtained from a healthy 26-year-old male volunteer, 174 cm tall and weighing 70 kg. A spiral three-dimensional CT scan of the pelvis was performed with a layer spacing of 0.6 mm, producing DICOM data, which were imported into Mimics 19.0 software for three-dimensional model reconstruction. Considering the prosthesis’s regular shape and incorporating existing research, geometric data were carefully measured, and certain parts were simplified. A three-dimensional solid model of the prosthesis was created using SolidWorks 2021 software. The assembly of bone tumor resection and prosthesis replacement was simulated, and both standard position prosthesis reconstruction models and models with different hip joint rotation centers were constructed. The finite element model was meshed and mechanically loaded using ProE Wildfire 5.0 and ANSYS 19.0, with seamless integration.

Setting the material properties of different structural components is a crucial step and fundamental requirement for finite element mesh division when constructing a finite element model [55]. In the pelvis, the stress on cortical bone is approximately 50 times greater than that on cancellous bone. Therefore, the pelvis is often simplified as composed entirely of cortical bone, as this assumption minimally affects the overall stress distribution [11]. Human bones exhibit anisotropy, meaning that stress and strain relationships in the longitudinal direction of cortical bone differ from those in the transverse direction, and the mechanical properties of cortical and cancellous bone vary across different body regions. Some researchers have modeled both cortical bone and prostheses as continuous, homogeneous, isotropic elastic materials, while others have considered the cortical bone to be anisotropic [56,57]. Comparative studies have shown that while anisotropic analysis is more complex, the differences in results compared to isotropic models are not significant [11,45,58]. This study, consistent with most literature, defines bone tissue as a continuous, isotropic, linear elastic material with fully bonded interfaces [59].

For finite element mesh division, solid elements are typically either tetrahedral or hexahedral. Tetrahedral elements can produce results that closely approximate theoretical values, while hexahedral elements offer greater stability in model analysis and are less affected by mesh simplification [60]. This study followed previous research practices by using tetrahedral-shaped solid elements for meshing the model [37,61]. The normal pelvic model consisted of 746,537 elements and 123,933 nodes; the hemipelvic prosthesis model contained 178,969 elements and 31,763 nodes; and the rotational center models post-prosthesis replacement, with different positions, included 631,376 elements and 109,347 nodes. When standing on both feet, the primary stress in the normal pelvis originated from the anterior S1 vertebra, passing through the S1 vertebra, bilateral sacroiliac joints, and iliac crest, and transmitted to the acetabular roof and both lower limbs. Specifically, the anterior S1 vertebra experienced 3.24 MPa of stress, the bilateral sacroiliac joints experienced 3.50 MPa, the iliac crest experienced 2.93 MPa, and the acetabular roof experienced 1.71 MPa, with symmetrical bone tissue stress on both sides. The stress on internal structures was relatively low, aligning with previous findings. This consistency indicates that the finite element prosthesis model is accurate, effectively analyzing static bone stress distribution and calculating stress magnitudes in the pelvis after semi-pelvic prosthesis assembly during standing.

When standing, the stress in the pelvis is primarily distributed around the sacroiliac joint, the arcuate line, the acetabulum, and the femoral head. The forces from the trunk are transmitted to the lower limbs through the axial bones [62]. Early finite element analyses of the pelvis, such as those by Vasue and Rapperport et al. [63,64], utilized two-dimensional data to establish their models. However, these models struggled to simulate the three-dimensional mechanical conduction mechanisms of the pelvis and were limited in their ability to fully analyze the mechanical characteristics of the pelvic ring across different planes. Goel et al. [65] developed a three-dimensional finite element analysis model of the pelvis but predominantly focused on the hip joint, often neglecting the stress distribution in the sacroiliac joint and the pelvic ring. As finite element research progressed, Anderson et al. [66] introduced a three-dimensional individualized design scheme for the pelvis. They demonstrated that variations in cortical bone thickness and elastic modulus could significantly influence pelvic stress distribution. This underscores the importance of considering individual patient characteristics in clinical practice and, if necessary, developing individualized models. Phillips et al. [67] advanced finite element modeling by incorporating the complete reconstruction of muscles and ligaments around the pelvis. Their 3D finite element model notably reduced stress concentration in both cortical and cancellous bone, offering a more accurate representation of the biomechanical characteristics of the living pelvis compared to previous models that only reconstructed the three-dimensional skeleton. This approach represents a significant advancement in finite element modeling by integrating muscular and ligamentous components.

Since surrounding ligaments and hip-circling muscles, along with their insertion points, were partially removed after pelvic tumor resection, the contraction directions of the reconstructed muscles would inevitably change. This alteration prevents an effective analysis of the biomechanics of the hip-circling muscles. Consequently, the current study model did not include the reconstruction of pelvic muscles and ligaments.

The stress distribution in both the normal pelvis model and the standard position prosthesis model during standing was primarily concentrated in the upper part of the sacral internal surface, the sacroiliac joint, the iliac crest, and the superior edge of the acetabulum. Zhou et al. [36] also confirmed this stress transmission pattern in the normal pelvis. The findings of the current study align with this distribution, showing that the internal side of the ischial ramus experiences less stress during standing compared to the pelvic ring biomechanics of the ischial tuberosity arch [68]. This stress distribution pattern is consistent with the normal physiological state of the pelvis, and the experimental results corroborate the actual situation. These results are in agreement with those of other researchers [11], thereby verifying the reliability and accuracy of the model.

From the stress distribution maps of all implant replacement models in the standing position, it is evident that stress concentration primarily occurred in the upper part of the sacral internal surface, the sacroiliac joint, the arch of the foot, the upper edge of the acetabulum, and the junction between the implant and the hip cup. Notably, the maximum stress value was observed at the connection between the implant and the hip cup.

For the standard replacement model, the stress distribution across the entire pelvic ring was relatively uniform, with no significant concentration at the ends of the implant. The near-end fixed screws and the smooth transition of the implant resection-bearing surface resulted in minimal stress at the fixation points of the pubic ramus and acetabulum, as well as the pubic ramus and the pubic fixation screw. This distribution closely resembled the stress characteristics of the normal model.

In the inward displacement model, stress concentration was noted in the upper part of the sacral internal surface, the sacroiliac joint, the arch line, the connecting rod, and both sides of the acetabular top. The stress distribution was similar to the standard model, with the maximum stress occurring in the connecting rod of the prosthesis (8.61 MPa). The stress in the bone-cutting bearing surface was lower than in the standard model. Still, maximum stress was observed at the distal end of the pubic bone, particularly at the fixation points of the pubic bone with the acetabulum, the pubic bone itself, and the pubic fixation screw. This suggests that the inward displacement model was prone to stress concentration in the pubic bone, resulting in higher stress values.

In the outward displacement model, the stress distribution was similar to the standard model, with only a slight increase in the stress value of the prosthesis compared to the standard model. The maximum stress value was found in the connecting rod of the prosthesis (5.36 MPa), while the stress in the bone-cutting bearing surface was 4.06 MPa—both lower than in the standard model. Compared to the inward displacement model, the outward displacement model exhibited a more uniform stress distribution, with generally lower stress values. This indicates that the outward displacement model’s stress distribution and magnitude were closer to those of the standard model, with a more even distribution and reduced stress values.

In the backward displacement model, the stress distribution was similar to the standard model, though the stress values were slightly higher. The maximum stress value occurred in the implant’s connection rod (9.31 MPa). The stress in the contralateral sacroiliac joint in the backward model was higher than in the ipsilateral joint, contrasting with the standard and other displaced models. The proximal and distal connection areas of the implant also showed significant stress increases, with the proximal longitudinal fixation screw exhibiting the highest stress among all models. This indicates a tendency for stress concentration at the contralateral sacroiliac joint, the connecting rod, and the proximal longitudinal screw in the backward model.

The stress distribution in the forward displacement model resembles that of the standard model, with only a slight increase in stress values. The maximum stress in the implant’s connection rod was 7.81 MPa. Although the stress distribution was similar to the standard model, there were minor differences in stress magnitude. Compared to the backward model, the forward displacement model showed a stress distribution and magnitude closer to the standard model, with a more uniform stress distribution and smaller overall stress values.

In the downward displacement model, the stress distribution was similar to the standard model, with a slight increase in stress values at the implant. The maximum stress in the implant’s connection rod reached 5.91 MPa, and the bearing surface of the resection was 3.98 MPa, both smaller than in the standard model. The stress distribution in other areas was relatively uniform, and compared to the upward displacement model, the downward displacement model showed lower maximum stress values. This indicates a more uniform stress distribution in the vertical direction, which helps avoid stress concentration and results in smaller stress values.

Therefore, the bone stress distribution after standard position replacement was relatively uniform, which supports the recovery of overall bone mechanics and aligns closely with natural bone stress conduction and distribution. This uniformity helps reduce stress concentration. When the hip joint rotation center deviated, although the stress distribution pattern of the pelvis remained similar, the stress magnitudes varied. Specifically, outward, forward, and downward deviations resulted in stress distributions and magnitudes similar to those in the standard position replacement model. However, when the implant connector rod and the proximal lateral fixation screw were displaced backward by 10 mm, the stress values reached their maximum across all models, indicating that backward displacement is likely to cause stress concentration in these components. Similarly, when the proximal central screw was displaced upward by 10 mm, it showed the highest stress value among all models, suggesting significant stress concentration at this location. Inward displacement of the implant by 10 mm resulted in stress concentration at three key locations: the distal end of the implant, where the acetabular fixation point and the ischial tuberosity were fixed; the ischial tuberosity itself; and the ischial fixation screw. These were the maximum values observed in all models, indicating that inward displacement is prone to causing stress concentration at the ischial tuberosity. Overall, compared to outward, upward, and forward displacements, inward, upward, and backward displacements were more likely to cause varying degrees of stress concentration in the pelvis and the prosthesis. Previous studies have indicated that the fatigue strength of cortical bone in the pelvis ranges from 120 to 150 MPa, while the fatigue strength of the modular hemi-pelvic prosthesis includes a tensile strength (σb) of 965 MPa for TC4 titanium alloy, a yield strength (σ0.2) of 905 MPa, and a tensile strength of 500 MPa with a yield strength of 405 MPa for TA2 titanium plate. The maximum stress values obtained in this study were significantly lower than these theoretical fatigue strengths, suggesting that the pelvis and prosthesis exhibit a high degree of safety and are less likely to experience fatigue or fracture when standing on both feet. However, this study did not account for stress distribution and magnitude during the gait cycle with prostheses, leaving the stress behavior of the prosthesis in daily activities undetermined. Thus, further research is needed to evaluate stress distribution during the gait cycle, particularly for inward, upward, and backward displacements.

The selection of a 70 kg volunteer for this study was based on this weight approximating the average body mass of patients treated in our clinical practice. While the study utilized this representative weight, the biomechanical conclusions are expected to be applicable to patients with both lower and higher body masses [69]. At the same time, the material mechanical properties of the implants in this study were assumed to be homogeneous, continuous, and isotropic. This assumption, while common in current finite element analysis models of bone, does not reflect the true anisotropic nature of real materials and represents an inherent limitation of this method [70].

In terms of modeling, this study did not simulate cancellous bone or reconstruct and analyze hip muscles, cartilages, intervertebral discs, and ligament tissues. The focus was solely on the biomechanical analysis of the semi-pelvic implant assembly used for reconstructing bone defects following pelvic tumor resection, specifically exploring the stress distribution of the semi-pelvic implant assembly with different hip joint rotation centers in the standing position of the lower limbs.

In a standing position, the pelvis bears no more than the weight of the patient [44]. During the gait cycle, the stress on the pelvis can increase significantly, reaching up to 4–7 times the patient’s weight on the side of the foot in contact with the ground and up to 10 times the weight of the patient during running and jumping [71,72,73]. Consequently, static stress analysis alone is inadequate for a comprehensive evaluation of prosthesis safety and durability. To accurately assess the stress distribution pattern of the pelvis with an implanted prosthesis in daily life, it is necessary to conduct analyses under gait cycle conditions and simulate specific actions performed by the patient.

Future research should aim to incorporate more realistic data by reconstructing the surrounding hip muscles, cartilages, intervertebral discs, and ligaments. Utilizing micro-CT to obtain the microstructure of cancellous bone and establishing a more precise finite element model will enhance the accuracy of the analysis. Comparative studies should then evaluate the stress distribution of the modular semi-pelvic prosthesis in various body positions (e.g., sitting, standing on one or two legs) under different loading states, walking speeds, and phases of the gait cycle. Such research will provide valuable insights and guidance for the clinical rehabilitation of patients undergoing such procedures.

## 5. Conclusions

The stress distribution and magnitude in the standard modular hemipelvic prosthesis model were comparable to those in the normal pelvis, confirming the prosthesis’s biomechanical compatibility. When the hip joint’s rotational center shifted within 10 mm, the stress distribution remained largely unchanged, suggesting that minor displacements do not significantly affect the pelvic load transmission in a bilateral standing position. Among the six tested displacement models, outward, forward, and downward shifts of the rotational center produced stress patterns closest to the standard model. In contrast, backward, upward, and inward shifts resulted in higher stress concentrations at specific points, particularly on the connecting rods and screws. These findings suggest that, in cases where some degree of rotational center displacement is unavoidable, an outward, forward, or downward shift is preferable to minimize stress concentrations and reduce the risk of prosthesis failure or component fracture.

## Figures and Tables

**Table 1:** Material parameters of the bone and modular hemipelvis prosthesis.

Material	Elastic Modulus (GPa)	Poisson’s Ratio	Fatigue Strength (MPa)
Titanium plate (TA2)	105	0.33	Tensile strength (σb) 500; yield strength (σ0.2) 425
Titanium alloy rod (TC4)	115	0.33	Tensile strength (σb) 965; yield strength (σ0.2) 905
Cortical bone	17	0.3	Superior and inferior pubic ramus 150; acetabulum 120

**Table 2:** The number of grids and nodes in each model.

Model	Number of Elements	Number of Nodes
Normal pelvic model	746,537	123,933
Hemipelvic prosthesis model	178,969	31,763
Model with different prosthesis rotation centers	631,376	109,347

**Table 3:** Maximum stress values at different locations of the pelvis in the normal pelvic model in the bipedal standing position.

Pelvic Location	Stress (MPa)
Anterior S1 vertebra	3.24
Sacroiliac joint	3.5
Arcuate line	2.93
Acetabular roof	1.71
Pubis ramus	0.005

**Table 4:** Stress distribution and maximum stress values of the prosthesis in the standard model.

Prosthesis Location	Stress (MPa)
Proximal central screw	3.11
Proximal transverse screw	3.2
Cortical bone-bearing surface	6.48
Connecting rod	7.26
Pubis and acetabulum fixation points	0.0028
Pubic ramus	0.0019
Pubic fixation screw	0.0035

**Table 5:** Maximum stress values at different locations of the pelvis in the standard and displacement model.

	Pelvic Location	Anterior S1 Vertebra	Sacroiliac Joint (Normal Side)	Sacroiliac Joint (Affected Side)	Acetabular Roof (Normal Side)	Acetabular Roof (Prosthesis)
Stress (MPa)						
Standard model		3.15	3.04	3.08	1.63	3.02
Inward displacement model		3.56	3.47	3.31	1.43	3.2
Outward displacement model		3.19	3.51	3.27	1.73	2.69
Backward displacement model		3.08	3.2	3.3	2.1	3.31
Forward displacement model		3.29	3.48	3.37	1.78	2.94
Upward displacement model		3.25	3.6	3.14	1.52	3.49
Downward displacement model		3.27	3.43	3.15	1.6	2.86

**Table 6:** Stress distribution and maximum stress values of the prosthesis in the inward displacement model.

Prosthesis Location	Stress (MPa)
Proximal central screw	4.96
Proximal transverse screw	2.39
Cortical bone-bearing surface	4.94
Connecting rod	8.61
Pubis and acetabulum fixation points	0.0676
Pubic ramus	0.0484
Pubic fixation screw	0.0513

**Table 7:** Stress distribution and maximum stress values of the prosthesis in the outward displacement model.

Prosthesis Location	Stress (MPa)
Proximal central screw	4.38
Proximal transverse screw	3.44
Cortical bone-bearing surface	4.06
Connecting rod	5.36
Pubis and acetabulum fixation points	0.0077
Pubic ramus	0.006
Pubic fixation screw	0.0074

**Table 8:** Stress distribution and maximum stress values of the prosthesis in the backward displacement model.

Prosthesis Location	Stress (MPa)
Proximal central screw	4.45
Proximal transverse screw	4.06
Cortical bone-bearing surface	5.32
Connecting rod	9.31
Pubis and acetabulum fixation points	0.0132
Pubic ramus	0.0091
Pubic fixation screw	0.0104

**Table 9:** Stress distribution and maximum stress values of the prosthesis in the forward displacement model.

Prosthesis Location	Stress (MPa)
Proximal central screw	4.05
Proximal transverse screw	3.23
Cortical bone-bearing surface	5.13
Connecting rod	7.81
Pubis and acetabulum fixation points	0.0089
Pubic ramus	0.0076
Pubic fixation screw	0.0103

**Table 10:** Stress distribution and maximum stress values of the prosthesis in the upward displacement model.

Prosthesis Location	Stress (MPa)
Proximal central screw	5.31
Proximal transverse screw	2.63
Cortical bone-bearing surface	7.14
Connecting rod	8.28
Pubis and acetabulum fixation points	0.0305
Pubic ramus	0.0085
Pubic fixation screw	0.0191

**Table 11:** Stress distribution and maximum stress values of the prosthesis in the downward shift model.

Prosthesis Location	Stress (MPa)
Proximal central screw	3.65
Proximal transverse screw	2.82
Cortical bone-bearing surface	3.98
Connecting rod	5.91
Pubis and acetabulum fixation points	0.0075
Pubic support	0.0029
Pubic fixation screw	0.0101

## Data Availability

The original contributions presented in the study are included in the article, further inquiries can be directed to the corresponding author.
